# Reply to Comments on ‘A functional polymorphism rs10830963 in melatonin receptor 1B associated with the risk of gestational diabetes mellitus’

**DOI:** 10.1042/BSR20200370

**Published:** 2020-02-28

**Authors:** Bo Huang, Yu-kun Wang, Lin-yuan Qin, Qin Wei, Nian Liu, Min Jiang, Hong-ping Yu, Xiang-yuan Yu

**Affiliations:** 1 Department of Epidemiology and Health Statistics of Guilin Medical University, Guilin 541100, Guangxi, China; 2 Department of Microbiology of Guangxi University, Nanning 530004, Guangxi, China; 3 Affiliated Tumor Hospital of Guangxi Medical University, Nanning 530021, Guangxi, China

**Keywords:** Gestational diabetes mellitus, MTNR1B, Polymorphism, meta analysis, Trial sequential analysis

## Abstract

Th authors of ‘A functional polymorphism rs10830963 in *melatonin receptor 1B* associated with the risk of gestational diabetes mellitus’ (*Bioscience Reports* (2019) **39**, 12) have written a reply in response to the correspondence piece by Rosta et al. (*Bioscience Reports* (2020) **40**, 2).

To the editor,

Many thanks to Professor Klara Rosta, M.D., Ph.D., Gábor Firneisz, M.D., Ph.D., *et al.* for their interest on our recently published article, ‘A functional polymorphism rs10830963 in melatonin receptor1B associated with the risk of gestational diabetes mellitus’ [1] and appreciate their comments [2] on it. We believe that peer exchanges among different research groups can promote better research works.

In the recent study, according to 14 reported research data on the association between a functional polymorphism rs10830963 in *MTNR1B* gene and the risk of gestational diabetes mellitus, we performed a meta-analysis by using Stata software, version 12.0 (Stata Corp LP, College Station, TX, U.S.A.) [3,4]. The false positive report probability (FPRP) analyses were adopted to confirm the positive findings [5,6]. Klara Rosta, M.D., Ph.D., *et al.* paid attention to one included study (good works from Rosta *et al.*, 2017) in this meta-analysis, then put forward some opinions and suggestions on the minor (rs10830963 G) allele frequencies (MAF), the calculation of effect value (odds ratios, ORs) and the indication of relevant clinical data (mean age and pre-pregnancy BMI). We are here to respond. If there are any inaccuracies in our response, we welcome to communicate again.

Since we read the original literature of Rosta *et al.*, 2017 [7], we found that not the exact genotyping data but an MAF of each studied SNP locus, including rs10830963 was reported. Therefore, we can not extract the accurate sample size data of being successfully genotyped. According to the number of 287 GDM cases meet the International Association of the Diabetes and Pregnancy Study Group (IADPSG) criteria and 533 controls reported in the literature, we estimated the genotype data by using the Hardy–Weinberg equilibrium (HWE) genotype distributions. The approach is recognized. As reminded by the commentary, we have carefully verified the extraction MAF in the literature, and hereby we correct it and other relevant research data.

We recalculate the results using the new genotype data, and the association between the SNP rs10830963 and the risk of GDM is still confirmed (Figures 1–3). Further functional experimental studies are warranted to explore and clarify the potential mechanism. Meanwhile, the variant rs10830963 G allele was estimated significantly associated with an increased GDM risk (CG vs. CC: OR = 1.44, 95% CI = 1.06−1.95; GG vs. CC: OR = 2.06, 95% CI = 1.26−3.37; G vs. C: OR = 1.44, 95% CI = 1.16−1.78) in the meta-analysis for Rosta *et al.*’s study data (Figures 1–3). There are slightly different of OR and corresponding 95% CI from the original literature. Maybe it was caused by meta-analysis process for different algorithms with stata software.

**Figure 1:**
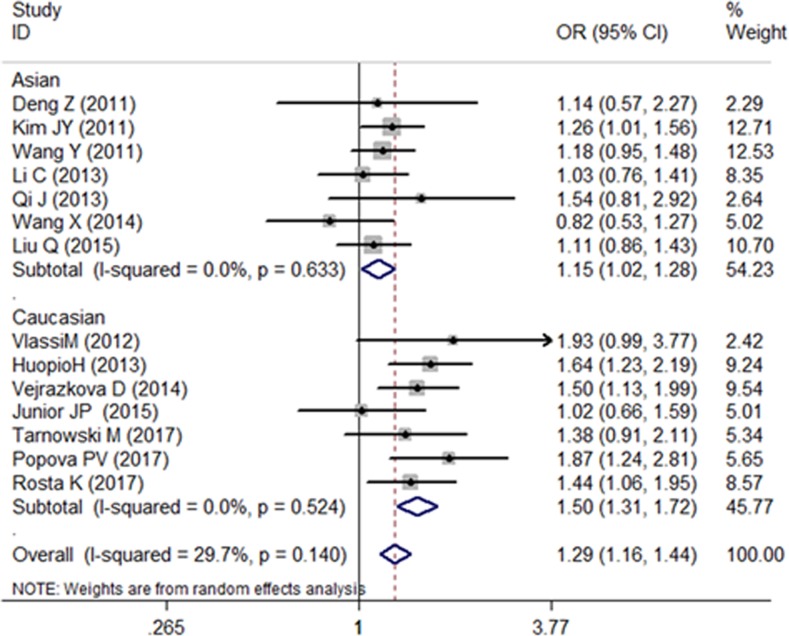
Forest plot on the risk of GDM associated with rs10830963 (CG vs. CC)

**Figure 2:**
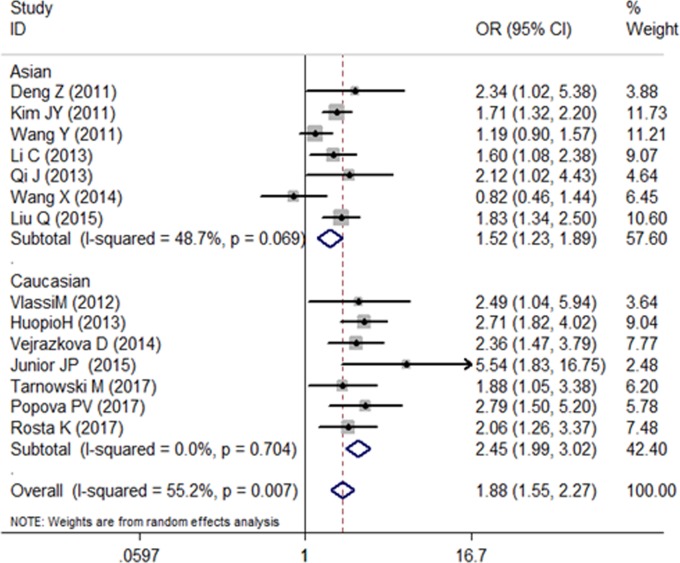
Forest plot on the risk of GDM associated with rs10830963 (GG vs. CC)

**Figure 3:**
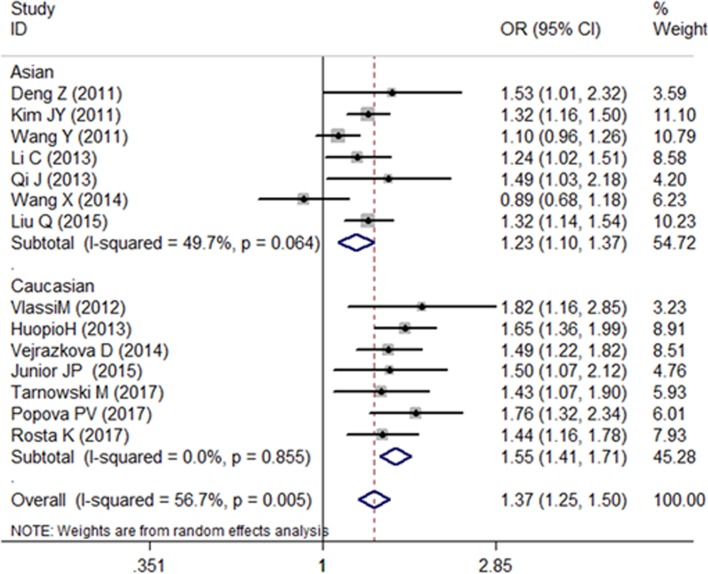
Forest plot on the risk of GDM associated with rs10830963 (G vs. C)

In epidemiological research, it is necessary to clarify the general demographic characteristics, and we have also carried out extraction and display in Tables 1–3. For the mean pre-pregnancy body mass index (BMI) and mean age values with the subjects, we have re-extracted and supplemented in the Table 1. The mean age of cases/controls were 32.04/30.51 in subjects of Austria and 33.70/31.25 of Hungary. Meanwhile, the mean BMI of cases/controls were 28.31/23.40 in Austria and 26.78/23.32 in Hungary (Table 1).

**Table 1:** Characteristics of the studies included in the meta-analysis

Author, year	Country	Diagnostic criteria	Genotyping methods	Controls	Number of case/control	MAF case/control	Mean age of cases/controls	Mean BMI of cases/controls	*P*_HWE_ for controls	NOS score
Deng Z., 2011	China	ADA	Sequencing	NGT	87/91	0.52/0.41	31.8 ± 4.6/29.7 ± 3.5	23.6 ± 3.0/21.5 ± 2.4	0.84	4
Kim J.Y., 2011	Korea	ADA	TaqMan	NGT	908/966	0.52/0.45	33.1/32.2	23.3 ± 4.0/21.4 ± 2.9	0.53	7
Wang Y., 2011	China	ADA	TaqMan	NGT	700/1029	0.46/0.43	30.0/32.0	21.5/21.7	0.81	8
Vlassi M., 2012	Greece	ADA	PCR-RFLP	NGT	77/98	0.41/0.28	35.4 ± 4.4/31.3 ± 5.2	25.8 ± 5.1/26.7 ± 6.2	0.02	4
Huopio H., 2013	Finland	ADA	Sequenom Assay/TaqMan	NGT	533/407	0.47/0.35	32.6/29.9	26.3 ± 4.7/24.1 ± 3.8	0.98	8
Li C., 2013	China	IADPSG	PCR-RFLP	NGT	350/480	0.45/0.40	32.4 ± 4.8/31.9 ± 5.2	25.3 ± 5.2/24.6 ± 4.6	0.79	8
Qi J., 2013	China	IADPSG	Sequencing	NGT	110/110	0.54/0.44	28.7 ± 3.1/28.1 ± 2.4	NA/NA	0.43	6
Vejrazkova D., 2014	Czech	WHO	TaqMan	NGT	458/422	0.38/0.29	34.1 ± 6.1/34.8 ± 15.1	24.3 ± 4.9/23.7 ± 4.2	0.48	8
Wang X., 2014	China	ADA	PCR-RFLP	NGT	184/235	0.42/0.45	28.2 ± 3.8/27.9 ± 4.1	21.2 ± 1.8/20.7 ± 1.4	0.53	6
Junior J.P., 2015	Brazil	ADA	real-time PCR	Healthy pregnant	183/183	0.28/0.20	32/29	32.0/25.4	0.11	7
Liu Q., 2015	China	ADA	TaqMan	NGT	674/674	0.51/0.44	31.6/32.1	24.4/25.2	0.02	8
Tarnowski M., 2017	Poland	IADPSG	TaqMan	NGT	204/207	0.39/0.31	31.7 ± 4.5/29.2 ± 5.0	25.1 ± 5.5/23.0 ± 4.0	0.112	7
Popova P.V., 2017	Russia	ADA	RT-PCR	Healthy pregnant	278/179	0.35/0.31	31.8 ± 4.8/29.4 ± 4.8	25.7 ± 5.9/22.9 ± 4.5	0.426	6
Rosta K., 2017	Hungary and Austria	IADPSG	KASP assay	Healthy pregnant	287/533	0.36/0.28	Hungary:33.70/31.25; Austria:32.04/30.51	Hungary:26.78/23.32; Austria:28.31/23.40	0.989	5

Abbreviations: ADA, American Diabetes Association; NGT, normal glucose tolerance; NOS, Newcastle–Ottawa Scale.

**Table 2:** Meta-analysis of the *MTNR1B* rs10830963 polymorphism on GDM risk

Subgroup	Heterozygous (CG vs. CC)				Homozygous (GG vs. CC)				Allele mogel (G vs. C)			
	Number of studies	Case/Control	OR (95% CI)	*P*_Effect_	Number of studies	Case/Control	OR (95% CI)	*P*_Effect_	Number of studies	Case/Control	OR(95% CI)	*P*_Effect_
Overall	14	3952/4736	1.29 (1.16–1.44)	<0.001	14	2628/2966	1.88 (1.55–2.27)	<0.001	14	10066/11228	1.37 (1.25–1.50)	<0.001
Ethnicity												
Asian	7	2271/2916	1.15 (1.02–1.28)	0.020	7	1543/1796	1.52 (1.23–1.89)	<0.001	7	6026/7170	1.23 (1.10–1.37)	<0.001
Caucasian	7	1681/1820	1.50 (1.31–1.72)	<0.001	7	1085/1170	2.45 (1.99–3.02)	<0.001	7	4040/4058	1.55 (1.41–1.71)	<0.001

**Table 3:** FPRP analysis for the significant associations of the *MTNR1B* rs10830963 C>G polymorphism and GDM risk

	OR (95%CI)	Prior probability					
		0.25	0.1	0.01	0.001	0.0001	0.00001
**Overall**							
CG vs. CC	1.29 (1.16–1.44)	0.002	0.005	0.056	0.375	0.857	0.984
GG vs. CC	1.88 (1.55–2.27)	0.002	0.007	0.070	0.433	0.884	0.987
G vs. C	1.37 (1.25–1.50)	0.001	0.004	0.038	0.286	0.800	0.976
**Asian**							
CG vs. CC	1.15 (1.02–1.28)	0.057	0.153	0.664	0.952	0.995	1.000
GG vs. CC	1.52 (1.23–1.89)	0.003	0.009	0.092	0.506	0.911	0.990
G vs. C	1.23 (1.10–1.37)	0.003	0.010	0.097	0.519	0.915	0.991
**Caucasian**							
CG vs. CC	1.50 (1.31–1.72)	0.002	0.007	0.074	0.446	0.889	0.988
GG vs. CC	2.45 (1.99–3.02)	0.016	0.047	0.351	0.845	0.982	0.998
G vs. C	1.55 (1.41–1.71)	0.002	0.005	0.056	0.375	0.857	0.984

Thank you very much again for Klara Rosta, M.D., Ph.D., Gábor Firneisz, M.D., Ph.D., *et al*.’s thoughtfulness and preciseness. Your comments means a great deal to us. Next, we will improve our study work together with the editors of ‘*Bioscience Reports*’.

## Abbreviations

BMI: body mass index
CI: confidence interval
FPRP: false positive report probability
GDM: gestational diabetes mellitus
MAF: minor allele frequency
OR: odds ratio
SNP: single nucleotide polymorphism
